# Supplementary material for: Tailoring the energy landscape of a bloch point domain wall with curvature
Source: Nat Commun. 2025 Aug 11;16:7422. doi: 10.1038/s41467-025-62705-x (PMC12339691; doi:10.1038/s41467-025-62705-x)
Supplement: Supplementary file 1 — Supplementary Information [file 41467_2025_62705_MOESM1_ESM.pdf]

Tailoring the energy landscape of a Bloch point domain wall with curvature

Sandra Ruiz-Gomez[1,2*], Claas Abert[3,4], Pamela Morales-Fernández[1], Claudia Fernandez-Gonzalez[1,2], Sabri Koraltan[2,3,5], Lukas Danesi[3,4], Dieter Suess[3,4], María Varela[6], Gabriel Sánchez-Santolino[6], Núria Bagués[2], Michael Foerster[2], Miguel Ángel Nino[2], Anna Mandziak[7], Dorota Wilgocka-Ślęzak[8], Pawel Nita[7,9], Markus Koenig[1], Sebastian Seifert[1], Aurelio Hierro-Rodriguez[10,11,12], Amalio Fernández-Pacheco[13], Claire Donnelly[1,14*]

[1]Max Planck Institute for Chemical Physics of Solids, 01187, Dresden, Germany

[2]ALBA Synchrotron Light Source, CELLS, Cerdanyola del Valles, 08290, Barcelona, Spain

[3]Faculty of Physics, University of Vienna, 1010 Vienna, Austria

[4]Research Platform MMM Mathematics-Magnetism-Materials, University of Vienna, 1010 Vienna, Austria

[5]Vienna Doctoral School in Physics, University of Vienna, Vienna, Austria

[6]Departamento de Física de Materiales & Instituto Pluridisciplinar, Universidad Complutense de Madrid, Madrid 28040, Spain

[7]SOLARIS Synchrotron light Sources, 30-392 Crakow, Poland.

[8]Jerzy Haber Institute of Catalysis and Surface Chemistry, PAC, 30-239 Krakow, Poland

[9]Faculty of Physics, Astronomy and Applied Computer Science, Jagiellonian University, 30-348, Crakow, Poland.

[10]Depto. Física, Universidad de Oviedo, 33007 Oviedo, Spain

[11]CINN (CSIC-Universidad de Oviedo), 33940, El Entrego, Spain

[12]SUPA School of Physics and Astronomy, University of Glasgow, G12 8QQ, Glasgow, UK

[12] Institute of Applied Physics, TU Wien, Wiedner Hauptstr. 8-10/134,1040 Vienna, Austria

[14]International Institute for Sustainability with Knotted Chiral Meta Matter (WPI-SKCM2), Hiroshima University, Hiroshima 739-8526, Japan

[*] Correspondence to srgomez@ucm.es or claire.donnelly@cpfs.mpg.de.

S1. XAS Spectra

In order to optimize the energy for maximum magnetic contrast, the X-ray Absorption Spectroscopy (XAS spectra) of Co was obtained from individual nanostructure by measuring stacks of PEEM images at different photon energies. After the normalization and alignment of images, the average of the signal in a selected region on the structure shadow is taken for each energy (Figure S1). Although the spectrum is noisy the line shapes is clearly those of metallic Fe with a main peak at the L3 edge and a wider peak at L2 with no evidence of oxidation.

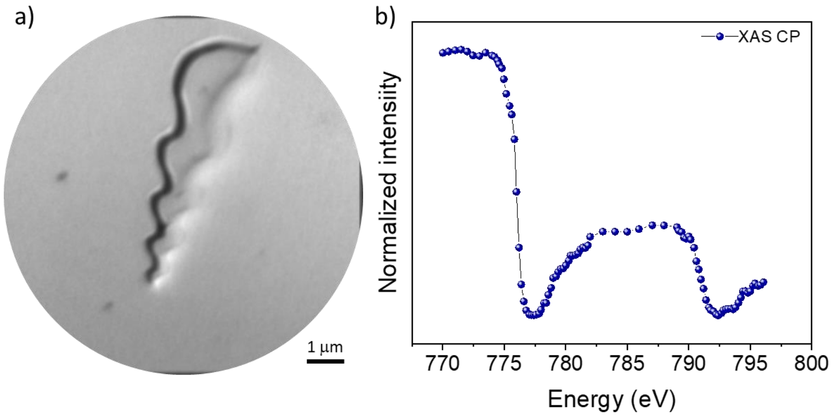

Figure S1: a) XAS image of an undulating structure measured at the Co L_3 edge. b) X-ray absorption spectrum of Co measured at the shadow of the nanostructure.

S2. Domain wall position under the application of magnetic fields

The Bloch point domain walls presented in this study were consistently located within the straight section between two curved regions. It is noteworthy to highlight the domain wall remains in this position under the influence of small magnetic fields as depicted in figure S2. Specifically, Figure S2a-c showcases X-ray Magnetic Circular Dichroism (XMCD) images of the shadow of a nanostructure that contain a domain wall in the position marked with a red arrow. Notably, the domain wall remains fixed in position despite the application of a magnetic field of 4 mT.

Given the challenges associated with acquiring XMCD images under magnetic fields, Magnetic Force Microscopy (MFM) images were obtained from the same nanostructure under a magnetic field of up to 20 mT. The domain wall that can be localize in the image by a strong white contrast, persists in its position, emphasizing that the straight section of the nanowires represents a well-defined local energy minimum.

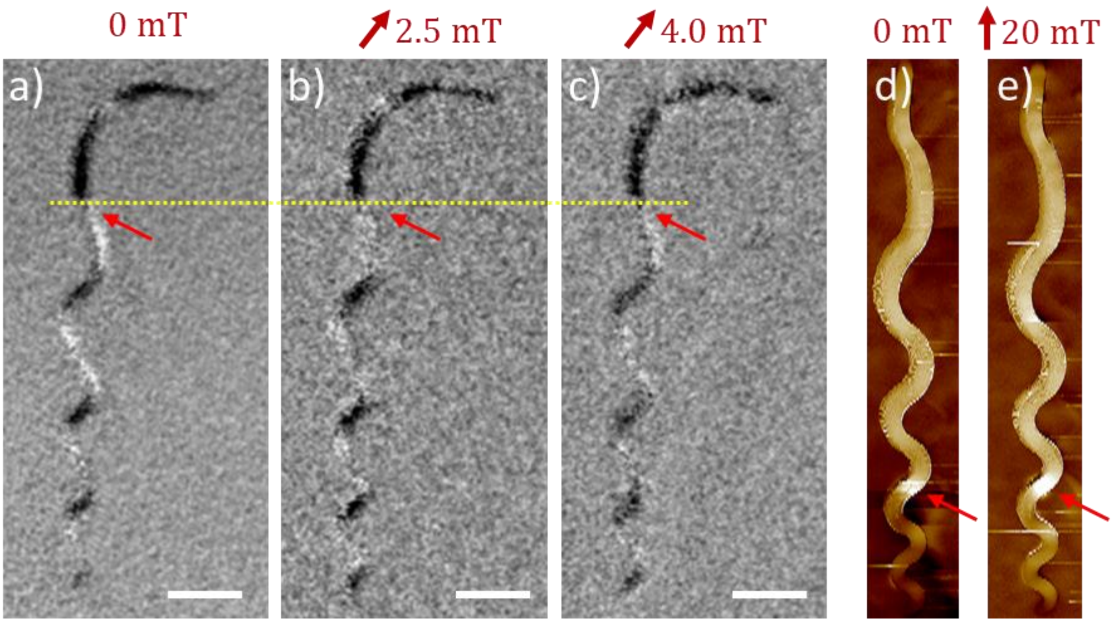

Figure S2: a) XMCD image measured at the Co L_3 edge for the structure shown in Figure 2 after the nucleation of a domain wall by applying magnetic fields parallel to the long axis of the nanostructure. The position of domain wall is denoted with a red arrow. b) and c) XMCD images of the same nanostructures, acquired with a magnetic field applied of 2.5 mT and 4.0 mT. d) MFM of the same nanostructures after the

nucleation of a domain wall (white contrast in the image). e) MFM image of the same nanostructures acquired with a magnetic field applied of 20 mT.

S3. Statistic of Domain wall depinning fields

A total of 29 observations of propagating domain walls were done in four different nanostructures, observing all of them pinned within the straight regions, corroborating our observation that the Bloch point exhibits a preference for non-chiral regions. Figure S3a shows the number of events for each radius of curvature studied. Figure S3b represent the component of the field perpendicular to the domain wall for the four nanostructures, showing a linear trend of the depinning field as a function of the curvature.

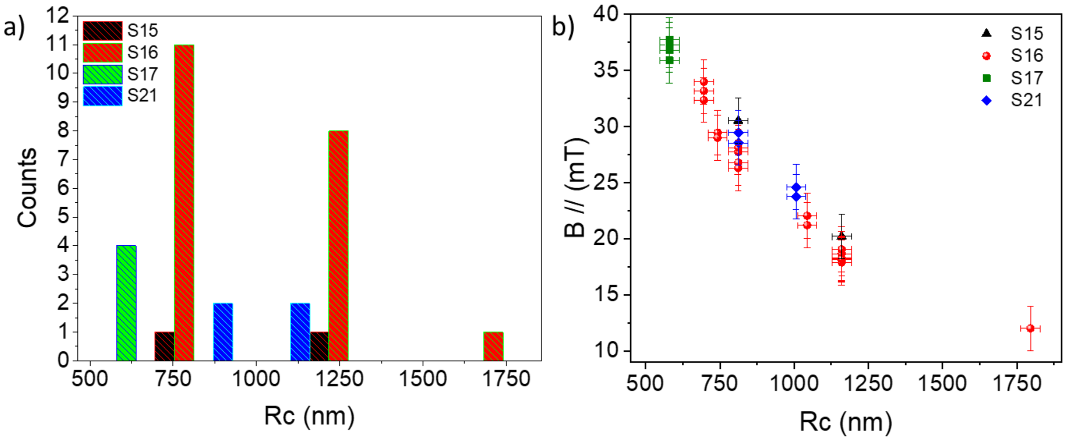

Figure S3: a) number of domain wall depinning experiments perform in 4 different nanostructures with different radius of curvature. b) Component of the magnetic field parallel to the wire as a function of the radius of curvature for four different nanostructures.

S4. Micromagnetic simulations of curved nanowires

The energies of the magnetization configurations in the converged transition path for a nanowire of 70 nm of diameter are shown in Fig. S4. Since the energy of a Bloch-point domain wall is dominated by the exchange contribution of the Bloch point, the numerical results are subject to significant noise caused by the irregular cell sizes in the tetragonal finite-element mesh despite the choice of 4 nm as a mesh cell size. This is due to the singular nature of the Bloch point that leads to a systematic underestimation of its exchange energy depending on the size of the containing simulation cell. In order mitigate this noise, the string simulation was repeated with 20 different tetragonalizations of the geometry. The resulting energies were averaged over the mesh realizations and this is the data shown in the figure.

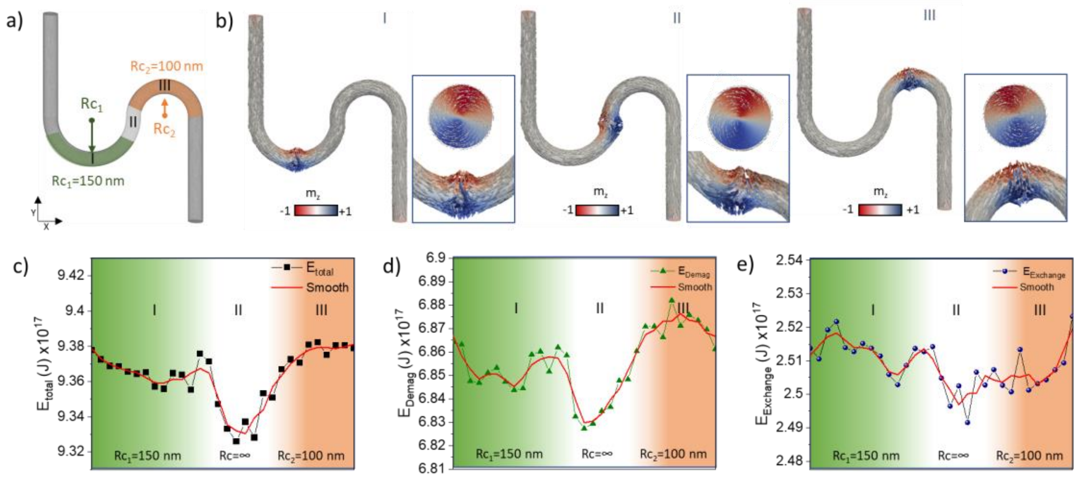

Figure S4**:** A schematic of the geometry is shown in a), with example positions of Bloch point domain walls shown in b. c) The energy landscape reveals a well-defined potential minimum in the straight region (II, white), with the total energy of the Bloch point domain wall increasing in regions of increasing curvature. d) The demagnetization energy reveals the same trend with well define minimum in the region II, not present in the exchange energy e).

S5. Micromagnetic simulations of curved nanotubes

In order to avoid the noise coming from the pinning of the Bloch point singularity on the mesh, a nanotube with a 70 nm diameter and an inner diameter of 15 nm was simulated. As shown in Figure S5, the data landscape is the same, showing the same trend as observed previously for the nanowire but with less noise. The plot is the result of one simulation without the need for averaging.

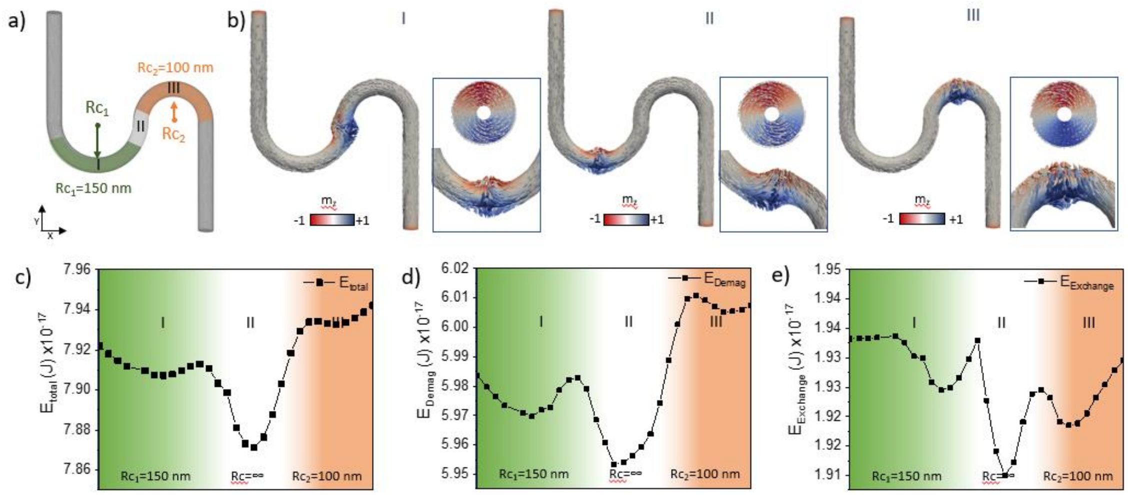

Figure S5**:** A schematic of the geometry is shown in a), with example positions of Bloch point domain walls shown in b. c) The energy landscape reveals a well-defined potential minimum in the straight region (II, white), with the total energy of the Bloch point domain wall increasing in regions of increasing curvature. d) The demagnetization energy reveals the same trend with well define minimum in the region II, also present in the exchange energy e). While the total and demagnetization energy is lower for higher radius of curvature, the opposite trend is observed for the exchange energy.

S6. Ratchet effect

The local curvature defines the energy barriers in our system, making possible to generate potential wells that are asymmetric due to neighbouring energy barriers of different heights, as predicted by the micromagnetic simulations (figure S4 and S5). This asymmetry would manifest as a non-reciprocity in the depinning fields of the domain wall, making it easier to propagate in one direction than in the other. We determine the asymmetry of the potential wells at the pinning points in the straight regions of the nanowire by measuring the depinning fields of the BPDW for both positive and negative field directions for four different starting position of the domain wall.

In figure S6, the starting point correspond to the images in the second column. The magnetic field positive (third column) or negative (first column) is applied until the domain wall moves. As can be seen, the depinning field needed to propagate the domain wall to the right is higher than the field needed to propagate the domain wall to the left.

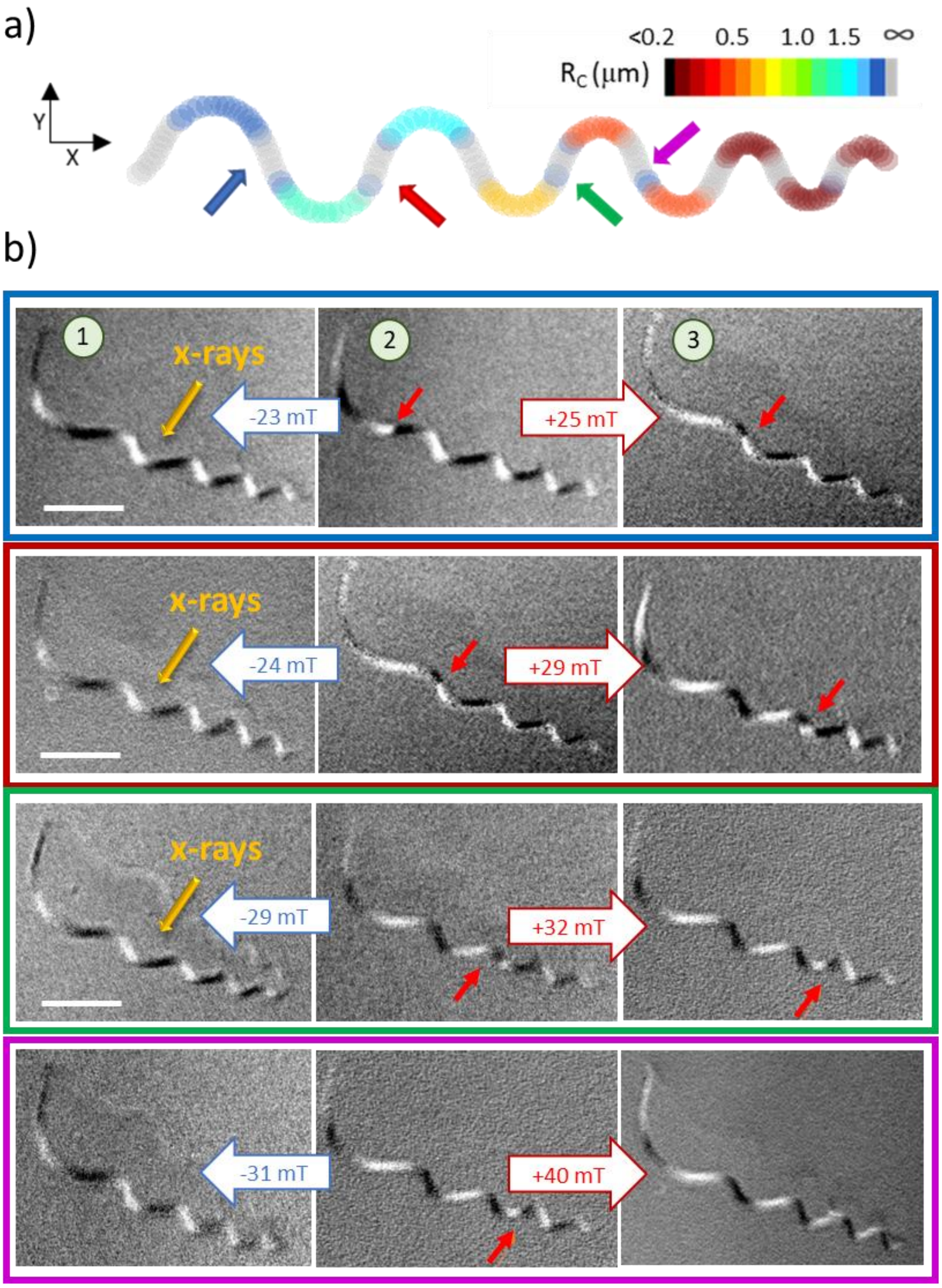

Figure S6**:** a) Schematic of the structure, where the color code indicates the radius of curvature along the structure, and the arrows mark the initial positions of the domain wall for the subsequent experiments.

b) XMCD images of the structure showing a domain wall pinned at a straight segment (2), and its propagation to the left (1) and to the right (3) after the application of magnetic field pulses of varying magnitude. The set of images in the blue box corresponds to the experiment starting with the wall at the first curvature arc. The red box corresponds to the second, the green box to the fourth, and the pink box to the fifth curvature arc. The scale bar is 1 µm in all images.

S7 Pinning Sources

Domain wall pinning is a critical phenomenon in nanowire-based devices that significantly affects their magnetic properties and dynamics under the application of current or magnetic fields. Although an ideal homogeneous monocrystalline nanowire with a perfect cylindrical shape would not exhibit DW pinning, such perfection is unachievable in practice. Various imperfections and defects can lead to DW pinning:

a) Shape Defects: Surface roughness, diameter modulations, or kinks and bends can change the local energy landscape, creating potential wells or barriers that trap domain walls.

b) Polycrystallinity and Associated Effects: The existence of grain boundaries and fluctuations in the crystallographic orientation of grains can create an inhomogeneous energy landscape, causing DW pinning at regions with different anisotropy energy.

c) Material Defects: Dislocations can introduce strain, and impurities or compositional inhomogeneities can change the local magnetic properties. These variations modify the energy landscape of the domain walls, generating pinning sites.

Diameter modulation

Domain walls are known to be sensitive to geometric and structural defects, which can lead to changes in the depinning field. To examine whether changes in diameter are a primary source of domain wall pinning, as well as their role in the formation of Bloch point domain walls in straight sections of nanowires the change of diameter along all the structures studied in this work is plotted.

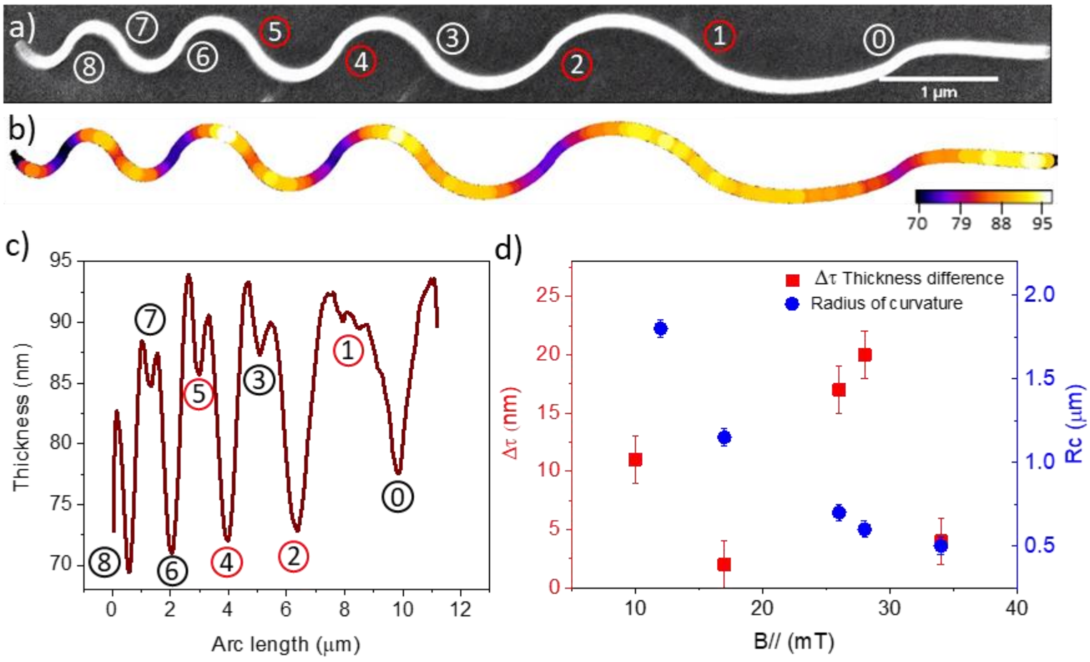

Figure S7**:** a) SEM image of the nanostructure, with numbered annotations indicating the straight regions within the structure. Red circles mark the locations where domain walls are pinned, while white circles denote regions where no domain walls are observed. b) Colour map displaying the diameter variation

along the nanostructure. c) Profile showing the changes in thickness (diameter) of the nanostructure, with numbers indicating the straight regions. d) Graph illustrating the depinning field as a function of both the radius of curvature and the change in thickness for the same nanostructure. A linear trend is observed for the depinning field required to depin the domain wall as a function of the radius of curvature, while no trend is observed for the change in diameter.

In figure S7 and S9 The SEM top view images are shown, indicating the straight regions with white numbers and the locations where domain walls were pinned with red circles. From the SEM images, the thickness/diameter variation along the nanostructure is obtained where the thickness is the diameter of the largest sphere that fits inside the object. This variation was color-coded to represent changes in thickness, with corresponding profiles graphically showing the thickness variation along the length of the nanowires.

While diameter changes can contribute to pinning, especially when the changes are substantial (e.g., figure S7 structures S16 at positions 2 and 4), pinned walls were also found in regions with minimal diameter variation (e.g., figure S7 structure 16 position 1 or figure S8 structure S17 at position 2 and S21 at position 3), indicating that diameter changes are not the primary source of pinning.

By plotting the depinning field as a function of the radius of curvature and the change in diameter for the different nanostructures, there is a clear linear dependence of the depinning field on the radius of curvature but no clear trend related to the slope of geometric defects. Indeed, when we calculate the correlation coefficient between the depinning field and the thickness, and curvature, we obtain correlation coefficients of [0.26] and [-0.94], respectively. These coefficients indicate that there is a much stronger correlation between the curvature and the depinning field, than the thickness, providing evidence that the depinning is dominated by the local curvature. These results suggest again that while diameter changes can contribute to pinning, they are not the dominant mechanism here.

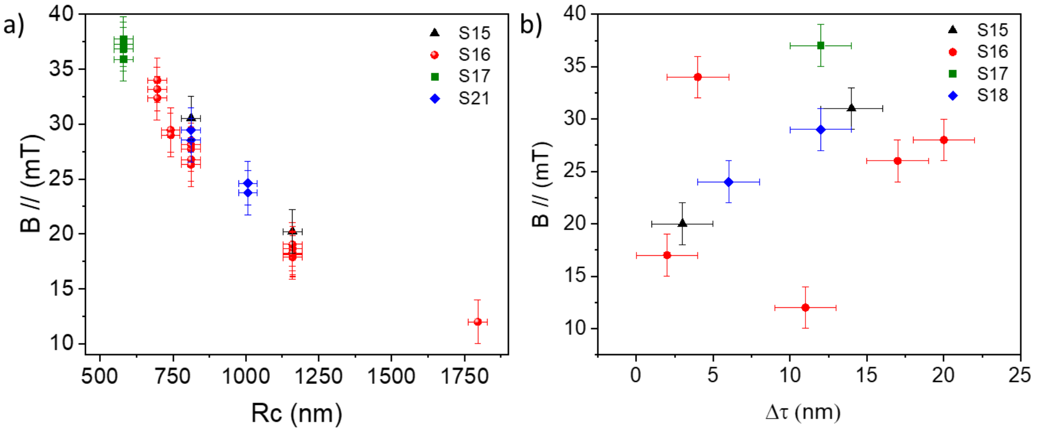

Figure S8: (a) Component of the magnetic field parallel to the wire as a function of the radius of curvature for four different nanostructures, where a clear linear trend can be observed. b) Component of the magnetic field parallel to the wire is plotted as a function of the variation in thickness for the same nanostructures, where no trend is observed.

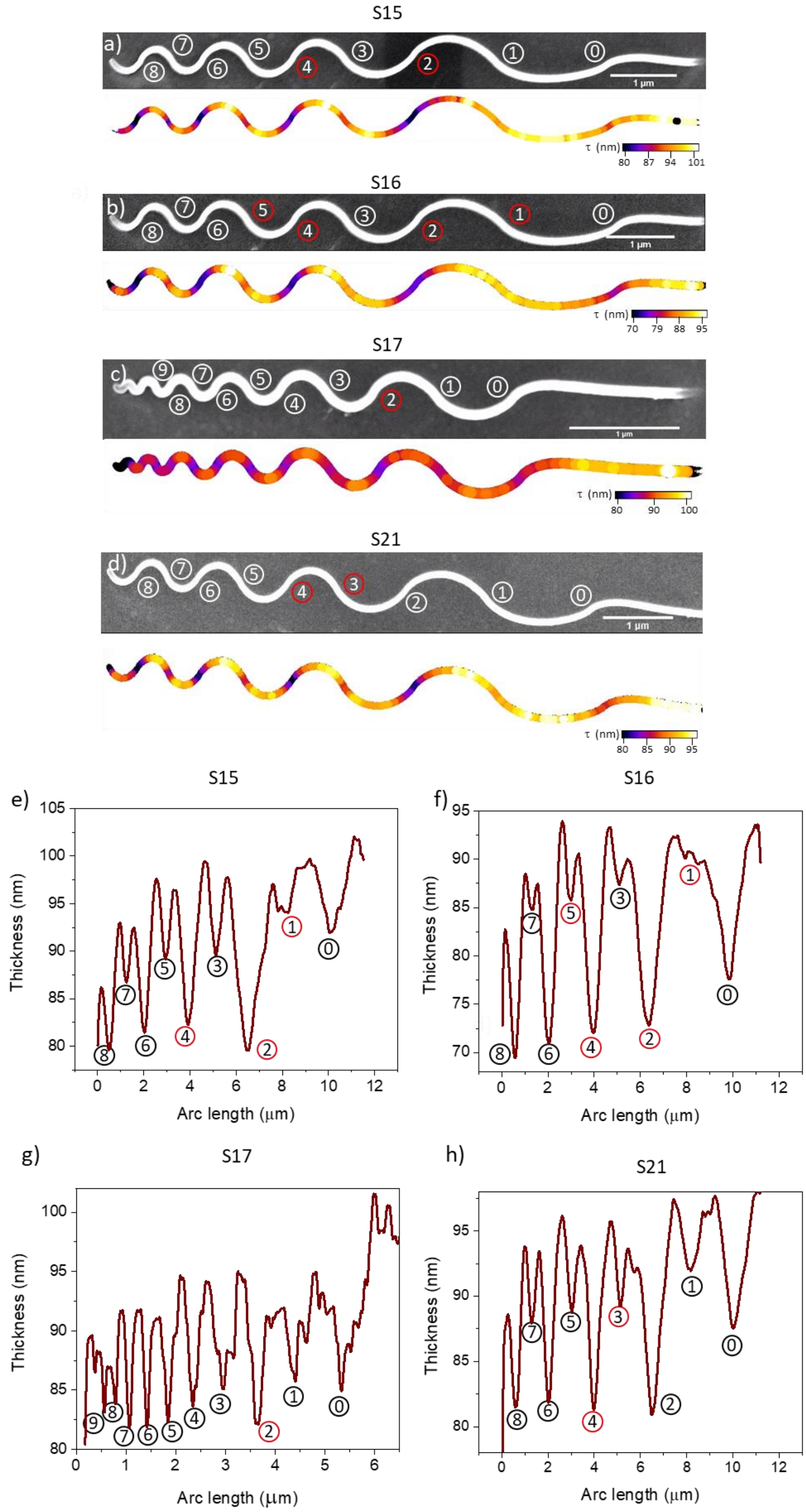

Figure S9**:** a, b, c, d) Scanning Electron Microscope (SEM) images of different nanostructures measured in this study, with numbered annotations indicating the straight regions within each structure. Red circles mark the locations where domain walls are pinned, while white circles denote regions where no domain walls are observed. Below the SEM images, color maps display the diameter variations along the nanostructures.e, f, g, h) Profiles showing the changes in thickness (diameter) of the previous nanostructures, with numbers indicating the straight regions.

Crystalline structure

To study the crystal structure and composition of the nanostructure, we performed aberration-corrected scanning transmission electron microscopy STEM and electron energy loss spectroscopy (EELS) mapping. The nanostructure under investigation was grown in a membrane between metallic contacts using the same growth conditions as the structure discussed in the main paper. To prevent the deposition of parasitic cobalt during growth, the membrane beneath the structure was removed.

Figure S10 presents high angle annular dark field (HAADF) and annular bright field (ABF) STEM images. In the first row, low magnification HAADF (bright contrast over dark background) and ABF (dark contrast over bright background) images are displayed, with regions marked by red circles corresponding to the high-magnification images shown below. These high-magnification ABF images, along with their respective fast Fourier transforms (FFT), reveal that the nanostructure is polycrystalline with small grain sizes. However, there is a noticeable orientation or texture, as parallel planes can be distinguished in the high-magnification images. The FFT analysis shows features corresponding to distances of 0.21 nm, 0.25 nm, and 0.29 nm. These planes were consistently visible along the nanostructures, with no differences observed between the curved and straight sections ruling out changes in the crystal structure of the nanostructures as a source of the pining of the walls. We note that the internal crystallinity of the nanostructure was better resolved in bright field contrast ABF images.

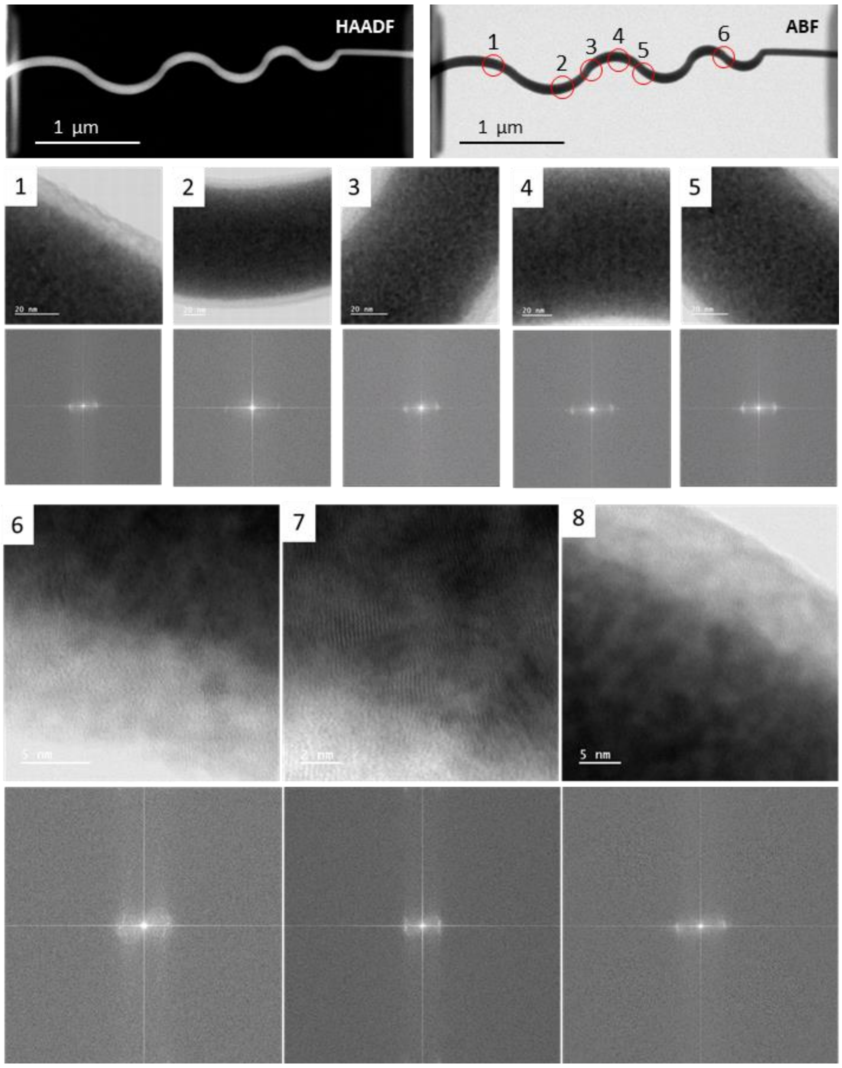

Figure S10: Low magnification HAADF and ABF images (upper row), indicating the regions where high magnification ABF images were acquired. High magnification ABF images 1-6 are displayed together with their corresponding FFT, below.

Composition.

Figure S11 illustrates the composition along the nanostructure. The first row displays a HAADF STEM image of the area where the electron energy loss spectrum image was acquired. The subsequent panels show the signals associated with the absorption edges of C *K*, O *K*, and Co *L*, respectively. In the color overlay, green represents the Co signal, red represents the O signal, and blue represents the C signal. Prior to generating signal maps, EELS spectrum was de-noised using multivariate statistical analysis (MSA) available in Gatan GMS software.

The analysis reveals that oxygen is primarily located on the surface of the nanostructure, effectively preventing the oxidation of the inner part of the nanowires. A layer of carbon is also observed on the surface, while only 2-3% of carbon is detected within the wire. Notably, there is no variation in composition along the length of the nanostructure, nor between the straight and curved sections, avoiding that compositional changes can be the origin of wall pining in straight regions.

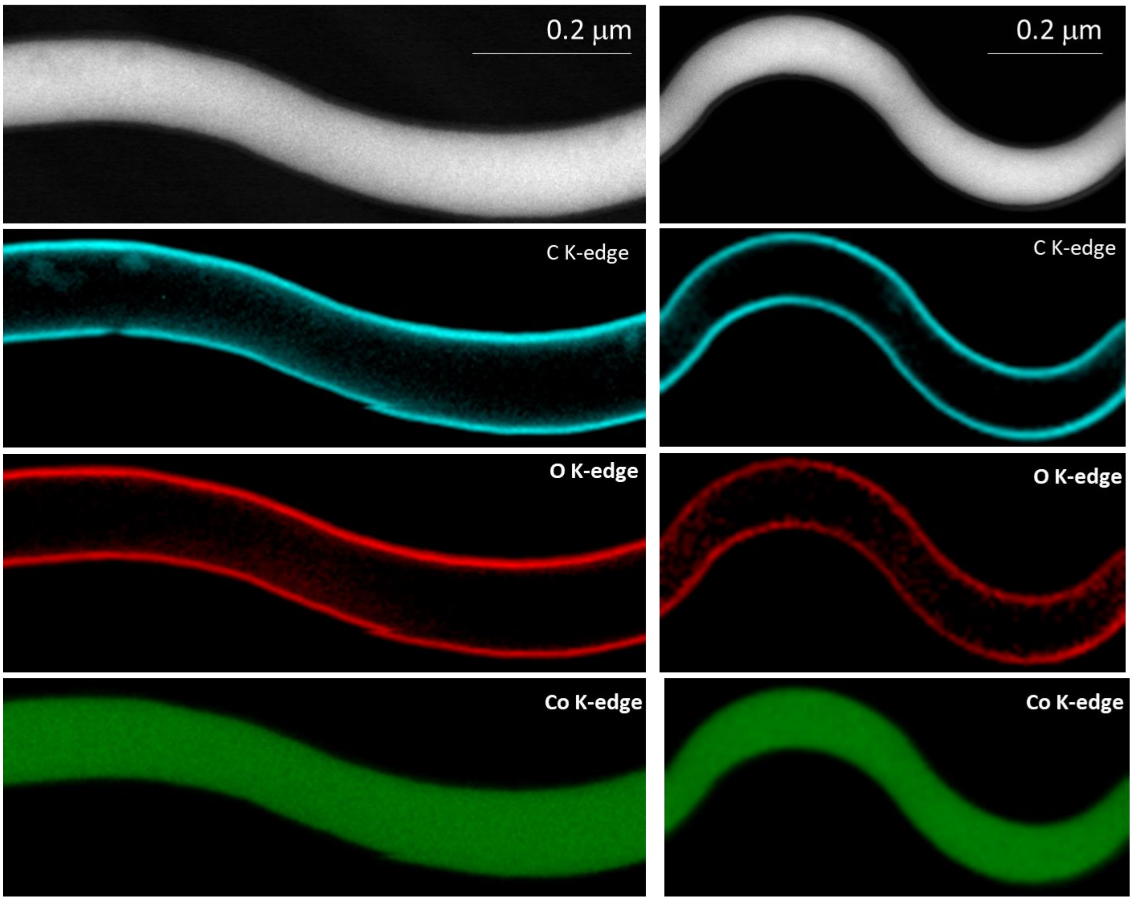

Figure S11: The first row shows the HAADF STEM image of the area where the EELS spectrum imaged was acquired. The following panels show the signals associated with the absorption edges of C K, O K and Co L respectively, with a colour overlay where green represents the Co signal, green represents the O signal and blue represents the C signal.
